# Atypical perigraft seroma masquerading as a forearm tumor in a dialysis patient

**DOI:** 10.5339/qmj.2022.20

**Published:** 2022-06-04

**Authors:** Samer Abdel Al, Mohamad K. Abou Chaar, Wafa Asha, Riyad T. Ellati, Riad Abdeljalil, Ayman Bitar, Muhamad Al-Qawasmi, Maysoun Hajir, Salam Elfarr

**Affiliations:** ^1^Department of Orthopedic Oncology, Hand, and Microsurgery, King Hussein Cancer Center, Jordan E-mail: Samer24al@yahoo.com; ^2^Department of Surgery, King Hussein Cancer Center, Jordan; ^3^Department of Radiation Oncology, King Hussein Cancer Center, Jordan; ^4^Department of Internal Medicine, Nephrology Division, King Hussein Cancer Center, Jordan; ^5^Department of Pharmacy, King Hussein Cancer Center, Jordan

**Keywords:** Perigraft seroma, soft tissue tumor, case report, and dialysis

## Abstract

Background: An extremely rare manifestation of perigraft seroma (PGS), in which a dense, semisolid jelly-like mass had formed around the shunt instead of the standard fluid-like form of the usual seroma, leading to misdiagnosis with other entities, such as tumors around the synthetic arterio-venous shunt (AVS) was presented.

Case Report: A 64-year-old male with multiple myeloma post autologous bone marrow transplant with a renal impairment, presented with a rare form of PGS, which was noticed 2 months after placing a synthetic AVS vascular graft. The mass increased in size, and multiple attempts for excision failed due to recurrence, which led to tumor misdiagnosis. The mass reoccurrence stopped completely only after the radical shunt removal.

Conclusion: This case report revealed a rare form of PGS, in which the seroma was represented as a firm, semisolid jelly-like mass rather than the typical fluid type transudate seroma. Despite its rarity, it was associated with a high recurrence rate because unlike the standard perishunt seroma, this semisolid jelly-like material could neither be aspirated, nor could it be resected en-bloc, leading to shunting dysfunction. Its management included advanced imaging and a high probability of shunt removal or replacement.

## Introduction

Perishunt seroma or perigraft seroma (PGS) is an unusual but often devastating complication of vascular surgery. This disease is manifested by a persistent, often enlarging, sterile fluid collection confined within a fibrous pseudo-membrane around a prosthetic graft.

Although such complications have been observed for several years, data regarding PGS are scarce, and information on this pathology is limited. Additionally, the majority of the literature is based on case reports, and no direct cause-effect or association has been established yet.

PGS has been linked to Blalock–Taussig shunts, synthetic dialysis catheters, and arterial bypass surgery.^1^ Given its ultrafiltrated serum-like content and surrounding soft tissue capsule, a clear etiology is yet to be determined.

Regardless of its unclear etiology, PGS is considered one of the most serious complications of polytetrafluoroethylene vascular grafts, resulting in dysfunction and thrombosis.^1^ It has been identified as a persistent sterile fluid collection confined by a fibrous capsule mostly in the mid-to-distal portion of the graft with an incidence rate of 1.7% to 4%.^2,3^

PGS can be clinically detected up to one year after insertion, with the highest incidence rate occurring during the first postoperative month after insertion, as reported in 25% of cases.^4^

Multiple hypotheses, ranging from graft handling and anastomosis to host cellular response, hemodynamic factors, and frequent puncture-related trauma have been proposed as possible causes of seroma, but none have been proven scientifically.^5,6^

The PGS gross pathology is usually composed of a glistening pseudocapsule encasing a sterile serous fluid collection around a prosthetic graft. Regarding the histology, it is mainly composed of mature fibroblasts with a rich collagen matrix forming the pseudocapsule and immature fibroblasts lining the outer graft with abundant chronic inflammatory cells, which are mostly giant multinucleated cells.^7^

Herein, a rare form of semisolid jelly-like component (rare variant of PGS) that necessitated the radical excision of the synthetic graft is presented. Documenting such rare presentations will raise the awareness for the differential diagnosis of non-vascular masses around synthetic shunts.

## Case Report

A 64-year-old male with hypothyroidism was diagnosed with light-chain multiple myeloma in 2003 and underwent multiple external beam radiation therapy sessions, followed by two cycles of vincristine 0.4 mg/adriamycin 18 mg/dexamethasone 40 mg and zoledronic acid 4 mg.

In December 2003, the patient underwent an autologous bone marrow transplant using the Melphalan protocol, which was complicated by graft-versus-host disease, which was managed with multiple lines of antimicrobial agents and corticosteroids.

The patient was kept on regular follow-up until 2011, when he was found to have lytic lesions at the 2^nd^, 5^th^, and 6^th^ thoracic vertebrae and cord compression at the tbl6 level, for which he underwent decompression and fixation followed by external beam radiation therapy.

In 2018, the patient was diagnosed with renal failure in Palestine and underwent left forearm antecubital arterio-venous shunt (AVS) procedure to start hemodialysis. Subsequently, the shunt was thrombosed, and another treatment was performed to implant the synthetic AVS (GORE-TEX^®^ Vascular Graft, W. L. Gore & Associates, Inc. Flagstaff, Arizona, USA) on the right forearm. The patient noticed a painless mass around the AVS on the right forearm almost 2 months later. The mass increased in size gradually until it reached 5 cm in diameter but without causing any symptoms other than some technical difficulties in accessing the shunt for hemodialysis. The mass was investigated by ultrasound, and it was reported as a non-vascular mass without any compromise to the venous or shunt systems. Therefore, an open biopsy was scheduled in June 2018.

The pathology was reported as granulation tissue in the form of fibroblasts and necroinflammatory debris. However, the possibility of malignant soft tissue tumor around the AVS could not be ruled out.

**Figure 2.**
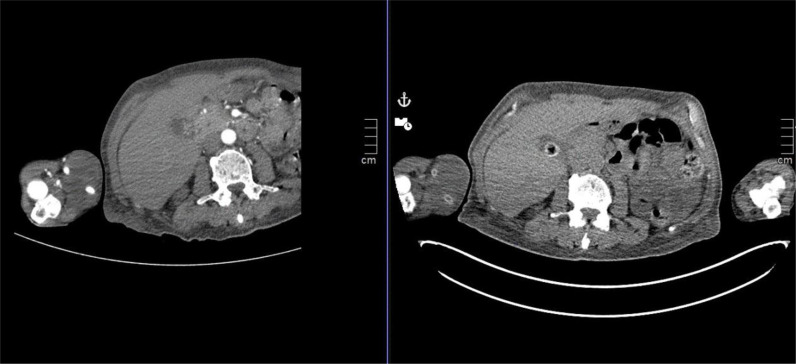
Computed tomography (CT) scan showing the mass. CT scan, axial view, and image with (left) and without (right) contrast of the right forearm and abdomen showing a recurrent non-enhancing soft tissue lesion at the antecubital fossa, measuring 6 cm in the craniocaudal dimension, and encasing the AVS without any definite vascular compromise nor evidence of associated bony destruction.

The patient was transferred to our orthopedic oncology department in late July of 2018 as a case of a possible tumor in the forearm (Figure. 1). Radiological investigation at our department started by computed tomography (CT) scan with and without contrast of the right forearm, which revealed a recurrent non-enhancing soft tissue lesion at the antecubital fossa, measuring 6 cm in craniocaudal dimension and encasing the AVS without any definite vascular compromise or evidence of associated bony destruction (Figure. 2). Positron emission tomography/CT scan showed multiple hypermetabolic bony and lymphatic lesions, indicating disease progression but without avidity to the forearm mass itself. Magnetic resonance imaging could not be performed due to the lack of material compatibility of the humerus nail, which was inserted outside our hospital for the right humerus fracture of the patient after he fell a few months before presentation.

A multidisciplinary medical decision was made to conduct an excisional biopsy of the mass while trying to salvage the AVS of the patient. The patient and his family were counseled about the procedure and signed consent.

**Figure 3.**
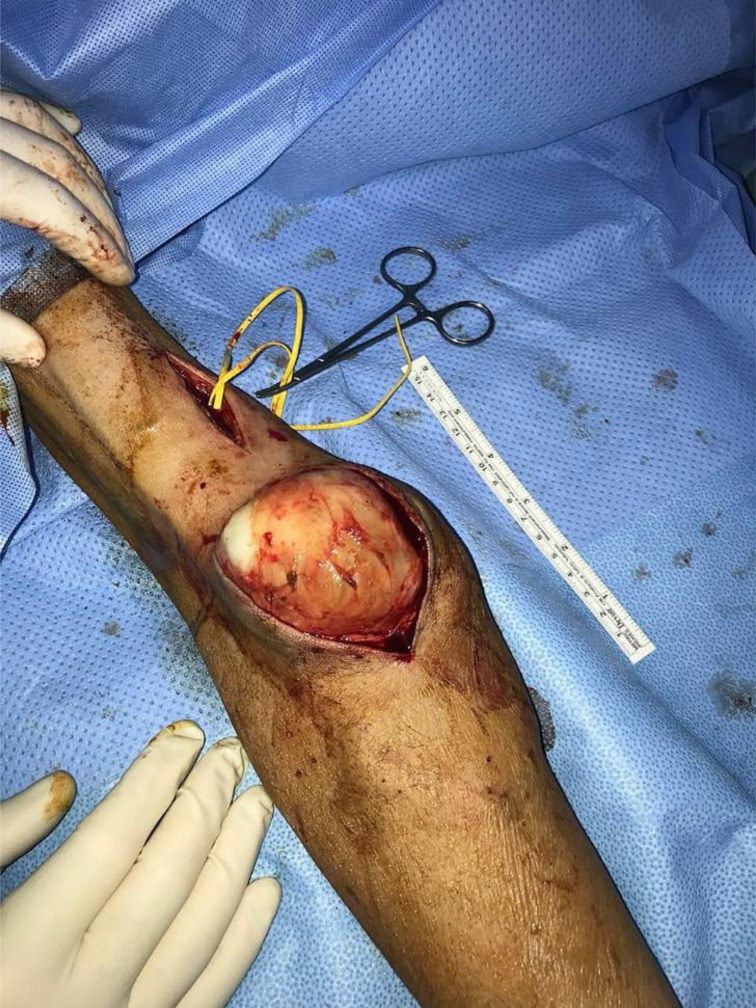
Exploration of the mass over the synthetic arterio-venous shunt.

The open biopsy started by a proximal control of the brachial artery and vein, followed by a longitudinal incision over the maximum bulge and with a careful dissection resulting in mass exposure (Figure. 3). Radical en-bloc excision was difficult due to the fragile texture of the mass. Thus, debulking of mass around this synthetic AVS was performed. Adequate hemostasis was attained followed by skin closure in layers. The procedure was followed by internal jugular dialysis catheter insertion as standby dialysis access in case that the AVS was not functioning.

The patient was discharged a few days later, and his first follow-up visit was 2 weeks later when he discovered a recurrence at the same site. The histopathological result came back again as fibro-adipose tissue with scattered multinucleated giant cells and necroinflammatory debris consistent with the atypical presentation of PGS. Thereby, another multidisciplinary clinic decision was made to proceed with radical excision of the mass along with his synthetic AVS.

**Figure 4.**
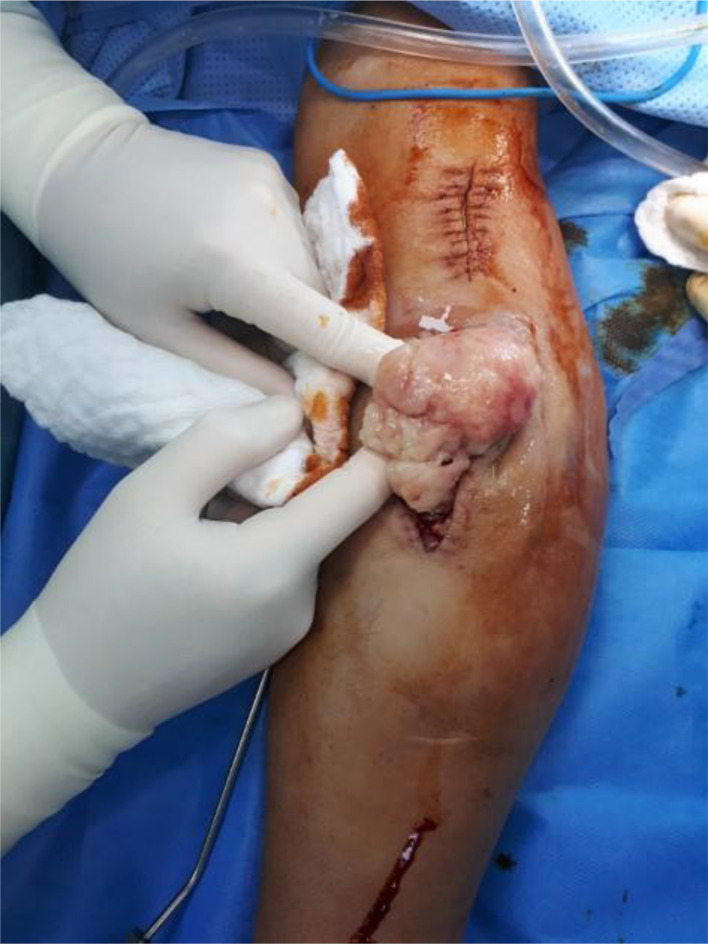
Intraoperative image showing the removal of the recurrent semisolid jelly-like tissue.

During the operation, excision of the recurrent tissue was attempted first (Figure. 4). Subsequently, we focused on the AVS removal, retaining less than 1 cm of graft stumps attached to the native vessels where they were closed using Prolene 5-0 suture. Next, an extensile longitudinal incision, connecting both of the previous proximal incisions, was utilized distally, which led to a better exposure. Finally, the abnormal tissues were completely removed until the superficial volar muscles were reached. All of the obtained gross materials along with the removed AVS were sent for permanent pathology, and special care was taken to preserve the important nerves, ensuring adequate hemostasis and closure over a drain.

**Figure 5.**
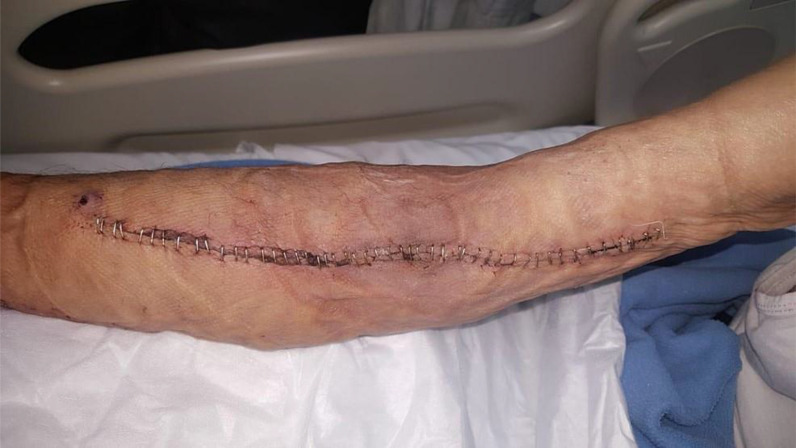
Postoperative image showing a well-healed wound without any signs of recurrence.

The patient was discharged on postoperative day 4; he was observed in the clinic almost a month after with no signs of recurrence (Figure. 5). The patient decided to continue his suggested treatment plan in his home country. The final pathology showed the same pathology, which was consistent with the semisolid jelly-like subtype of PGS. During regular virtual follow-up, the patient experienced no recurrence, until he passed away in December 2019 due to myocardial infarction.

## Discussion

The incidence of PGS is rare, and the best treatment regime remains debatable.

PGS detection is difficult due to its frequently asymptomatic nature. Hence, any persistent fluid accumulation around the graft for more than one month is deemed abnormal.^7^

Such PGS is more frequently encountered in upper arm grafts compared with forearm grafts due to the higher flow rate in the former. Multiple theories have been proposed to explain the increased frequency in upper arm grafts. Such findings are mostly attributed to the increased subcutaneous fat liquefication resulting from tissue dissection during graft insertion.^4,8^

Diagnosis is mainly clinical, and based on a high level of suspicion, the fluid around AVS can indicate an acute infection, hematoma depending on blood acuity, abscesses, and less occasionally PGS. Despite the importance of blood tests to exclude infection, color Doppler ultrasound imaging is usually the first choice to evaluate graft patency and PGS size.

Computed tomographic angiography can provide more secondary details to differentiate seroma from hemorrhage.^9^

Although the etiology of PGS remains unclear, some preoperative factors, including anticoagulation medication, smoking, and diabetes have been suggested as risk factors for PGS formation.^9^

Potential complications of PGS include intractable pain, sac rupture, and graft compression or thrombosis.^10^

Such complications lead to multiple modalities for the management of PGS. Various treatment strategies are considered for PGS; these strategies include the cessation of anticoagulation therapies, percutaneous aspiration, surgical capsule resection, graft replacement, and application of microfibrillar collagen around the grafts.^7,9^

The absolute treatment for refractory PGS is graft excision or replacement, as opposed to the repeated percutaneous drainage which has been proven ineffective with the potential to increase the risk of graft infection.^11,12^

When necessary, graft replacement with a different material is the most definitive treatment, and can include a decrease in graft permeability and periprosthetic reaction to a foreign body.

Despite the absence of an established difference between Dacron and expanded polytetrafluoroethylene (PTFE) replacement,^9^ recent studies indicate a strong association of PGS presence with the use of PTFE grafts.^13^

Pseudocapsule removal and surgical drainage should be avoided because they are not curative and cause a high rate of infection.^4^ Alternative therapies that have been tested in a small number of cases include replacement with homografts,^14^ native vein,^15^ saphenous vein wrapping,^16^ interposition of covered stents,^17^ and microfibrillar collagen injection into the periprosthetic space.^18^

Plasmapheresis and intravenous fibrinogen administration have been used to eliminate hypothetical serum factors, which are thought to alter normal graft permeability.^6,19^

More recent animal studies revealed that radiation-crosslinked gelatin hydrogel of the prosthetic vascular graft can mimic the chemical and physical properties of the extracellular matrix in vivo, may aid in wound healing, and may effectively prevent serious sequelae, such as seroma formation and infection.^20^

In this case study patient, multiple surgical excisions were deemed unsuccessful due to technical challenges encountered in the removal of the semisolid jelly-like tissue, leading to the rapid recurrence. All the findings indicated the need for radical excision of the shunt to achieve resolution.

Finally, exemption should be applied to sarcomas in solid non-vascular masses around AVS, notably epithelioid angiosarcoma, where almost all malignant endothelial cells are epithelioid in appearance. A total of 54% of the angiosarcomas arising from dialysis AV fistulas have the histological features of epithelioid angiosarcomas.^21–27^

## Conclusion

Non-vascular masses around synthetic AVS in dialysis patients are considered a challenging entity, and can be serious and lead to shunt dysfunction. Although the differential diagnosis can be shortlisted with the histopathology of the biopsy, the findings can still be misleading due to the rare encounter of this semisolid jelly mass. Preservation of the shunt may not be an option to avoid recurrence.

### Future Recommendation

PGS is a rare presentation for a very common procedure (dialysis shunt). The most successful method in handling recurrent PGSs is the surgical excision of the AVS. This method has been proven to be the most effective, specifically in rare semisolid jelly-like types as presented in our case report. Documenting such rare presentations will raise the awareness for the differential diagnosis of non-vascular masses around synthetic shunts.

### Declarations

Ethical approval and consent to participate:

NA

### Consent For Publication

Written informed consent was obtained from the patient‘s next of kin for publication of this case report and any accompanying images. A copy of the written consent is available for review by the Editor-in-Chief of this journal.

### Availability of Data and Material

NA

### Funding

NA

### Competing Interests

The authors declare that they have no competing interests.

### Acknowledgments

NA

## Figures and Tables

**Figur 1.**
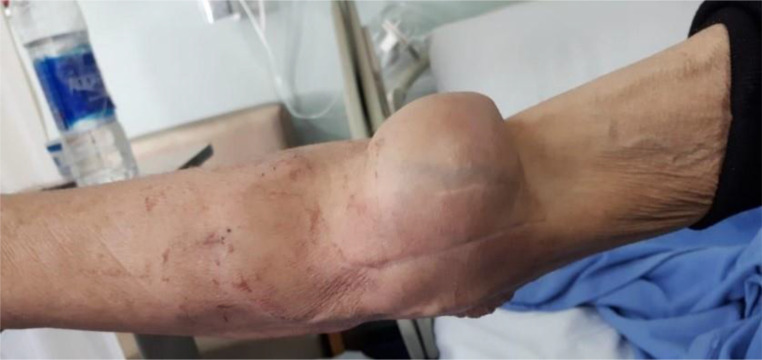
Initial presentation with a firm forearm mass around the synthetic arterio-venous shunt.
